# Virus-induced gene silencing as a tool for functional genomics in weeds: Challenges and future directions

**DOI:** 10.1590/1678-4685-GMB-2025-0252

**Published:** 2026-08-14

**Authors:** Érika Frydrych Capelari, Márcia Margis-Pinheiro, Aldo Merotto, Rogerio Margis

**Affiliations:** 1 Universidade Federal do Rio Grande do Sul, Departamento de Genética, Porto Alegre, RS, Brazil.; 2 Universidade Federal do Rio Grande do Sul, Departamento de Plantas de Lavoura, Porto Alegre, RS, Brazil.; 3 Universidade Federal do Rio Grande do Sul, Departamento de Biofísica, Porto Alegre, RS, Brazil.

**Keywords:** Virus-induced gene silencing, VIGS, RNAi, functional genomics

## Abstract

Virus-induced gene silencing (VIGS) has evolved from a conceptual demonstration of antiviral defense into a pivotal reverse-genetics platform for plant functional genomics. By exploiting engineered DNA- or RNA-based viral vectors, VIGS enables rapid, sequence-specific transcript knockdown through RNA-mediated degradation of target transcripts. Recent refinements in vector design, inoculation strategies, and viral species selection, such as TRV, BSMV, and FoMV, have expanded its application to previously recalcitrant plants, including major crops and emerging weed models. In weeds, functional genomics remains particularly challenging due to high genetic variability, limited genomic resources, and incompatibility with conventional viral vectors and transformation systems. In this context, VIGS provides a tractable approach to investigate genes associated with herbicide resistance, metabolic adaptation, and stress tolerance. Beyond weed biology, its application to studies of immune signaling, hormonal crosstalk, and secondary metabolism highlights VIGS as a versatile biotechnology for elucidating gene function and supporting next-generation strategies in plant improvement and integrated pest management.

## Introduction

Although plant viruses are traditionally regarded as pathogens that negatively impact plant health and crop productivity, advances in biotechnology have transformed them into valuable molecular tools (Wang et al., 2020). The first demonstrations of Virus-induced gene silencing (VIGS) were performed using the phytoene desaturase (PDS) and green fluorescent protein (GFP) genes, employing TMV and PVX-based systems (Kumagai et al., 1995; Ruiz et al., 1998). Since then, VIGS has become a widely used reverse genetics approach that employs the natural antiviral defense machinery of plants to achieve sequence-specific downregulation of endogenous genes. VIGS exploits engineered plant viruses either through direct plant inoculation or via infection mediated by modified *Agrobacterium tumefaciens* strains, which deliver viral genomes into plant cells (Zulfiqar et al., 2023). 

Plant viruses represent obligate parasites, as they are entirely dependent on their host for replication, lacking intrinsic machinery for protein biosynthesis or energy production (Boualem et al., 2016). Their genomes, composed of either single- or double-stranded DNA or RNA, typically encode only a limited set of genes, encapsulated within a protein shell termed the capsid (Chaitanya, 2019). Viral entry into host cells cannot occur autonomously; instead, it requires breaches in plant tissue or transmission by biological vectors (Whitfield and Rotenberg, 2015). 

By modifying viral genomes to carry short fragments of plant genes, the host defense system targets both the viral RNA and the corresponding endogenous transcripts, leading to efficient and transient suppression of the gene of interest (Dommes et al., 2019). Beyond its basis in antiviral defense, VIGS has emerged as a powerful experimental tool for plant functional genomics, enabling rapid generation of loss-of-function phenotypes without the need for stable transformation. This makes the system particularly attractive for both model and non-model plant species, including crops and weeds that are otherwise recalcitrant to genetic manipulation (Ramegowda et al., 2014; Rössner et al., 2022). Taken together, VIGS stands out as a rapid, efficient, and adaptable system for functional analysis of plant genes. Its ability to transiently suppress gene expression in a sequence-specific manner, combined with its scalability and broad applicability across taxa, ensures its continued importance in advancing plant molecular biology and biotechnology. In this review, we provide an overview of the virus-induced gene silencing (VIGS) approach, emphasizing its mechanism and experimental implementation. We further highlight how VIGS has been applied to investigate gene function in a wide range of plant species, from classical model organisms to non-model and crop species, and briefly discuss its emerging use in functional studies of weed species.

## VIGS: From the first functional vector to a key strategy for functional genomics

The first functional VIGS vector was developed by Kumagai et al. (1995), using Tobacco Mosaic Virus (TMV) in the model plant *Nicotiana benthamiana* (Kumagai *et al.,* 1995). The efficient silencing of the Phytoene Desaturase (PDS) gene expression was obtained by inserting a fragment of the NbPDS gene into the viral genome and inoculating the *in vitro* transcribed RNA into plant leaves, producing a characteristic albino phenotype (Kumagai *et al.,* 1995). This achievement validated the principle of virus-mediated gene silencing and established VIGS as a practical tool for gene functional studies. 

**Figure 1 -:**
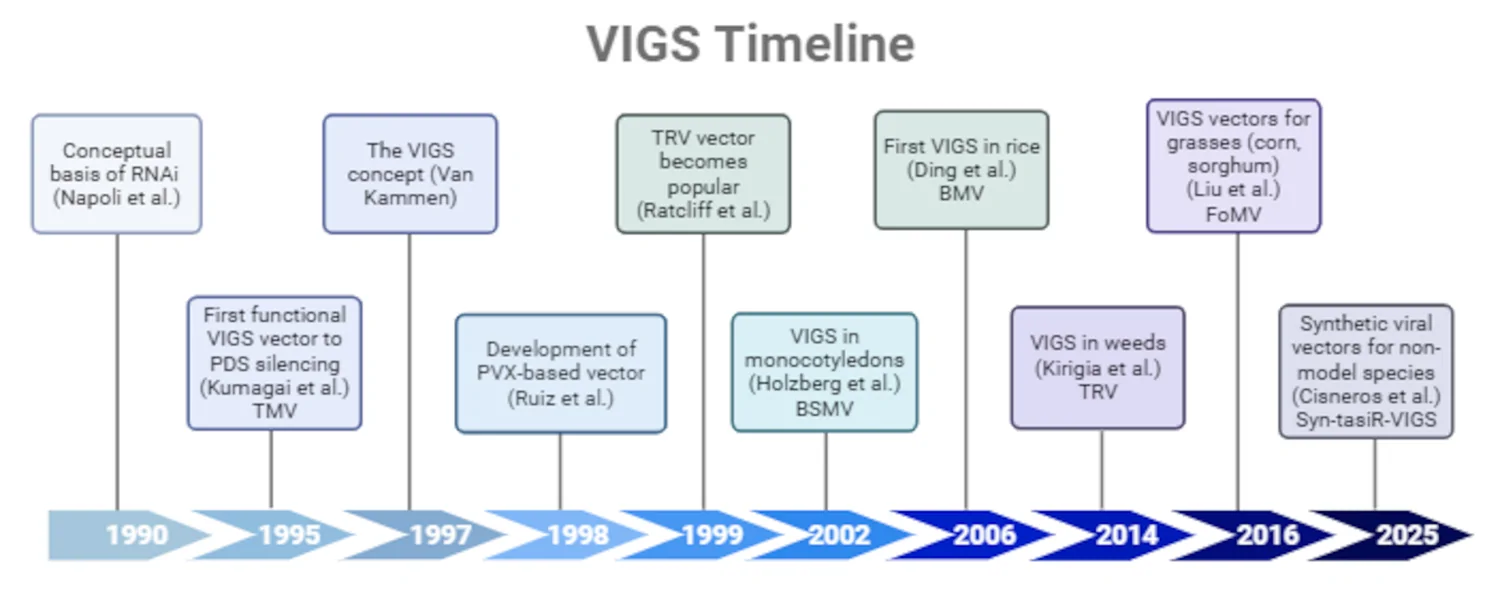
Timeline summarizing the VIGS evolution, highlighting breakthroughs, methodological improvements, and its expanding use in diverse plant species. List of references: Napoli et al., 1990; Kumagai et al., 1995; Van Kammen, 1997; Ruiz et al., 1998; Ratcliff et al., 1999; Holzberg et al., 2002; Ding et al., 2006; Kirigia et al., 2014; Liu et al., 2016 and Cisneros et al., 2025.

In 1997, the concept of VIGS was first introduced by Van Kammen, who used the term to describe the phenomenon of “recovery from viral infection”, referring to the ability of plants to reduce viral load and symptoms over time, gradually restoring normal growth following the initial infection (Van Kammen, 1997). Over the years, the methodology has been increasingly applied and refined, becoming a versatile and powerful tool for functional genomics (Figure 1). Continuous improvements in viral vectors and inoculation strategies have expanded its use across a wide range of plant species, including both model and non-model systems, thereby enhancing its relevance in plant biology and crop research (Zaulda et al., 2022; Akher et al., 2025).

VIGS rapidly became a key strategy for functional genomics research because of its unique advantages over traditional genetic approaches. Unlike stable transformation, which is time-consuming and species-dependent, VIGS enables the generation of knockdown phenotypes within a few weeks, often 2-3 weeks post-inoculation (Kumagai et al., 1995; Ratcliff et al., 1997; Ruiz et al., 1998), allowing researchers to directly associate specific genes with their biological roles. The transient and high-throughput nature makes VIGS ideally suited for large-scale gene function screening. Moreover, its applicability to a wide range of plant species has positioned it as a highly flexible and adaptable platform in plant biology (Bernacki et al., 2010; Zhang et al., 2010).

The analysis of gene functions in recalcitrant plant species for genetic transformation has advanced considerably through virus-induced gene silencing. VIGS is useful across a broad phylogenetic spectrum of plants. In dicots, it has been widely applied in species such as *Arabidopsis thaliana*, *Nicotiana benthamiana*, tomato, and soybean, while in monocots it has been successfully adapted to important crops including barley, rice, wheat, and maize (Holzberg et al., 2002; Scofield and Nelson, 2009; Lee et al., 2012; Mei et al., 2016). This cross-species applicability highlights the robustness of the approach and reinforces its relevance for both fundamental research, aimed at understanding gene function, and potential use in applied research, such as crop improvement, stress tolerance, and the development of biotechnological tools (Burch-Smith et al., 2004; Kalia et al., 2024). In particular, the ability of VIGS to circumvent the challenges of stable transformation makes it especially valuable for high-throughput functional genomics in species with limited genetic resources. Additionally, VIGS is generally transient, but in a few cases can be transmitted to the next generation as well. For example, in *Nicotiana benthamiana* and tomato, VIGS was shown to persist for over two years and to be inherited by progeny seedlings, indicating long-term and heritable gene silencing, providing a new dimension for the application of VIGS in plants (Senthil-Kumar and Mysore, 2011). Nevertheless, it is still unclear whether such heritable silencing can also occur in weed species.

Despite limited data in weeds, the same methodology holds promise for functional genomic studies in these species, providing a foundation for exploring gene functions associated with growth, adaptation, and herbicide resistance.

## The process behind the system

**Figure 2 -:**
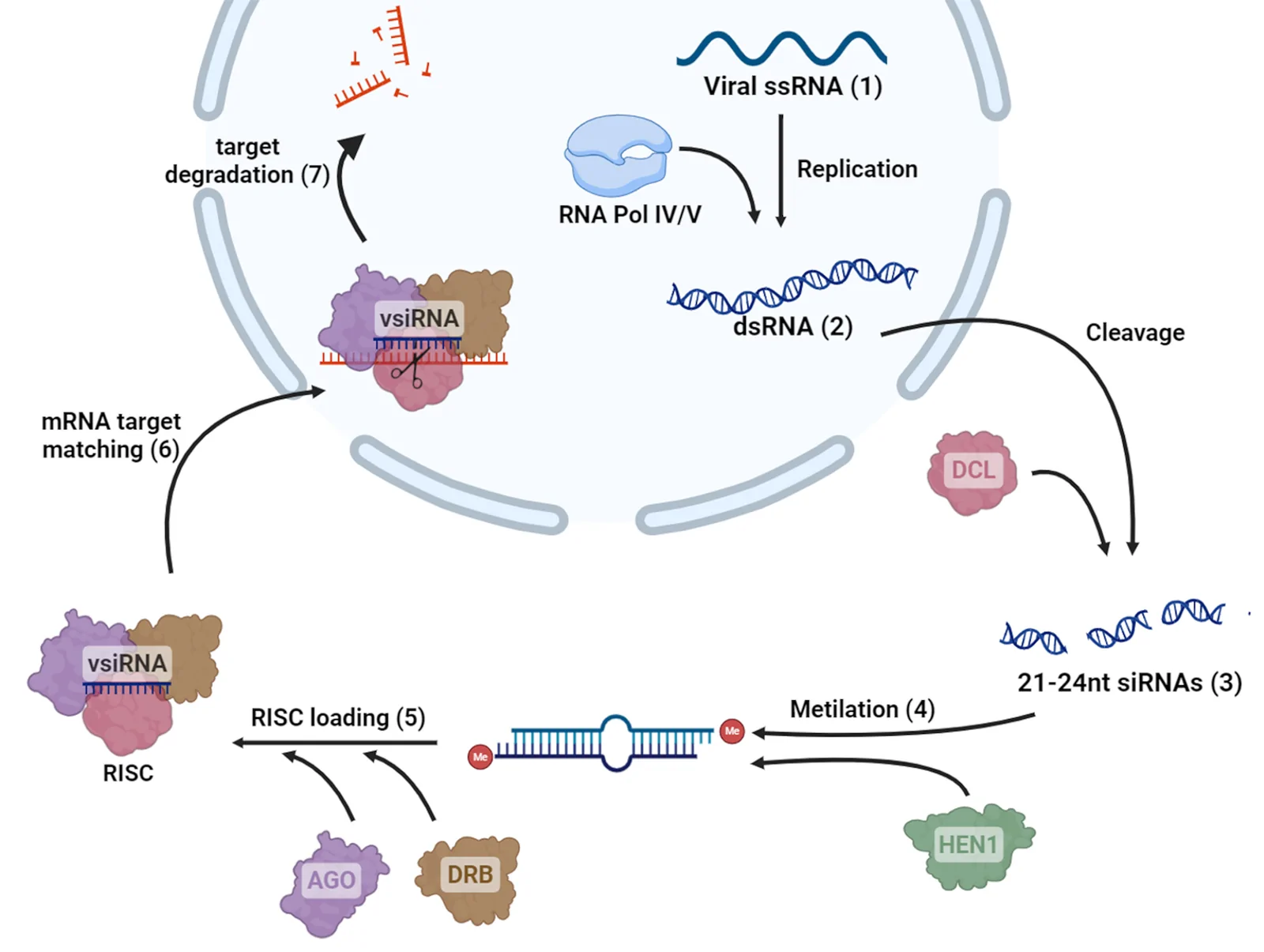
Schematic overview of the RNA silencing mechanism triggered by virus-induced gene silencing (VIGS), exemplified by a DNA virus. Once inside the cell, recombinant viral transcripts (1) are converted into double-stranded RNA intermediates (2) by the activity of RNA-dependent RNA polymerase. Dicerlike (DCL2 or 4) proteins recognize and cleave these dsRNAs into 21-24 bp small interfering RNAs (siRNAs) (3). After the duplex separation, each siRNA duplex is methylated by the 2′-O-methyltransferase HEN1 (4), and the guide strand is loaded onto Argonaute (AGO 1, 2, or 5) proteins by DRBs (2,3, or 4) while the passenger strand is degraded. The resulting AGO-siRNAs complex is subsequently incorporated into a multi-protein RNA-induced silencing complex (RISC) (5), which specifically targets and binds to RNA sequences complementary to the guide strand (6), leading to the degradation of both viral RNAs and endogenous transcripts sharing sequence homology (7).

At the mechanistic level, the technique operates through the host RNA interference (RNAi) pathway, grounded in the observation that many RNA viruses activate a conserved RNA silencing pathway. The virus enters the mesophyll cells, and once inside, its genome is replicated (Figure 2). Typically, for DNA viruses, replication occurs in the nucleus (Charman and Weitzman, 2020), whereas for RNA viruses, it takes place in the cytoplasm (Hull, 2014), where the viral RNA serves directly as mRNA and is translated by the plant ribosomes. Recombinant viral transcripts, whether viral transcripts (for DNA virus) or genomic RNA (for RNA virus), containing plant gene fragments are converted into double-stranded RNA intermediates, with the assistance of RNA-dependent RNA polymerase (RdRP). RNase III-like enzyme known as Dicer-like (DCLs) recognizes and cleaves these dsRNAs into double-stranded small interfering RNA (siRNA) of 21-24 base pairs. DCL2 and DCL4 proteins are directly involved in the antiviral defense system against RNA viruses (Ding and Voinnet 2007). After the duplex separation, double-stranded RNA-binding proteins (DRB 2, 3, or 4) bind to the guide strand, and then this complex is loaded onto Argonaute (AGO 2,3,5,7, or 10) proteins, while the passenger strand gets degraded. This AGO-siRNA complex is subsequently incorporated into a multi-protein RNA-induced silencing complex (RISC), where it guides the sequence-specific degradation of both viral RNAs and endogenous transcripts sharing sequence homology (Burch-Smith et al., 2004; Unver and Budak, 2009). The same mechanism is used by the VIGS system. 

## VIGS vectors and inoculation strategies

**Figure 3 -:**
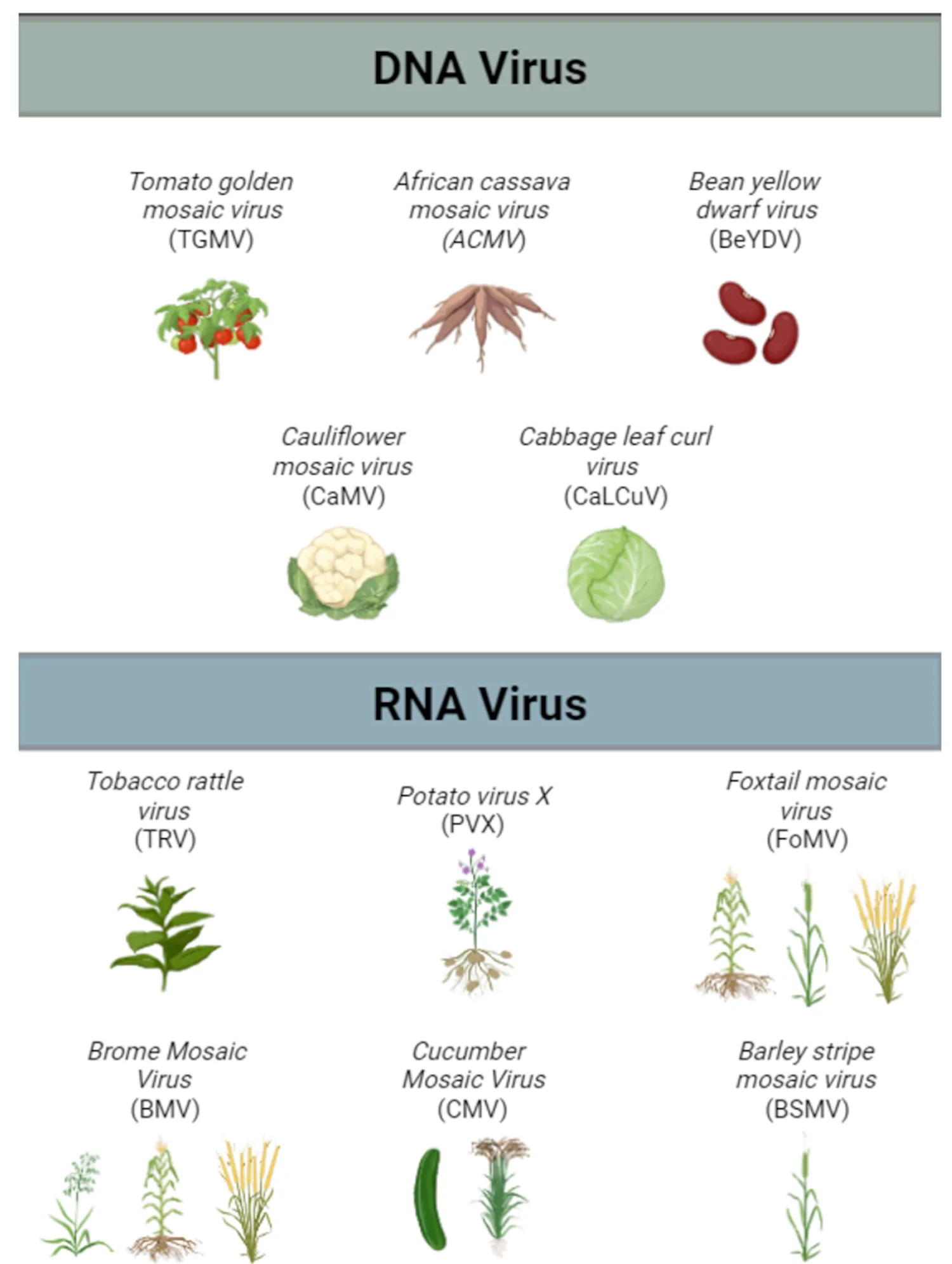
Overview of viruses employed in virus-induced gene silencing (VIGS) systems. The upper panel illustrates DNA viruses previously used in VIGS studies and their known host plants, whereas the lower panel presents RNA viruses and their associated host species. Tomato Golden Mosaic Virus (TGMV), African Cassava Mosaic Virus (ACMV), Bean Yellow Dwarf Virus (BeYDV), and Cabbage Leaf Curl Virus (CaLCuV) belong to the Geminiviridae family. Cauliflower Mosaic Virus (CaMV) belongs to the Caulimoviridae family. Tobacco Rattle Virus (TRV) and Barley Stripe Mosaic Virus (BSMV) are from the Virgaviridae family. Potato virus X (PVX) belongs to the Alphaflexiviridae family. The Foxtail Vosaic Virus (FoMV) is from the Alphaflexiviridae family, whereas the Brome Mosaic Virus (BMV) and Cucumber Mosaic Virus (CMV) belong to the Bromoviridae virus family.

Engineered viral vectors have been extensively characterized and repurposed not only for gene silencing, but also for heterologous gene expression and genome engineering applications. Their versatility has proven particularly useful for functional studies in both model and non-model organisms, as highlighted in recent reviews (Kant and Dasgupta, 2017; Cody and Scholthof, 2019; Abrahamian et al., 2020; Rössner et al., 2022). These advancements underscore the potential of viral vectors as central tools in plant molecular biology. VIGS vectors can be classified into either DNA-based-VIGS or RNA-based-VIGS (Figure 3).

DNA-based VIGS: Although most VIGS systems rely on RNA viruses, several DNA viruses, particularly geminiviruses, have been successfully adapted as VIGS vectors (Nagar et al., 1995; Lu, 2003). Geminiviruses are small, single-stranded DNA viruses with twinned icosahedral particles and bipartite or monopartite genomes. Their ability to replicate via the rolling-circle mechanism and to spread systemically makes them attractive tools for functional genomics (Zerbini et al., 2017; Bonnamy et al., 2023). For example, Bean yellow dwarf virus (BeYDV) and Cabbage leaf curl virus (CaLCuV) have been developed into VIGS vectors in dicot plants such as *Arabidopsis thaliana* and *Nicotiana benthamiana*. Inside host cells, DNA viruses replicate through a process that involves delivery of the viral genome into the nucleus, where viral and host enzymes coordinate the synthesis of the new viral DNA (Fay and Panté 2015). After uncoating, the viral DNA serves as a template for transcription of viral mRNAs by host RNA polymerase, which are then translated into viral proteins in the cytoplasm. The viral DNA replication can follow different strategies depending on the virus type, such as the rolling-circle mechanism used by geminiviruses or bidirectional replication in larger DNA viruses. These DNA-based systems expand the applicability of VIGS to hosts that are less susceptible to RNA virus infection, making them valuable tools for functional genomics in diverse plant species.

RNA based VIGS: The majority of VIGS vectors employed for VIGS are derived from RNA, for two main reasons: i) due to their low molecular weight and high infection penetrance (Unver and Budak, 2009), and ii) RNA viruses can trigger silencing in various host plants, like *Capsicum frutescens*, *Arabidopsis thaliana*, *Nicotiana benthamiana*, cotton plants, Solanaceae, and many other monocotyledon plants, such as *Oryza sativa*, *Hordeum vulgare*, and *Zea mays*, to eliminate endogenous transcripts (Kant and Dasgupta, 2017). Single-stranded RNA (ssRNA) viruses can be positive-sense (+ssRNA) or negative-sense (−ssRNA). Their replication within a host cell occurs through a highly coordinated process involving the synthesis of a complementary copy of the viral genome (Rampersad and Tennant 2018). For +ssRNA viruses, the viral RNA functions directly as messenger RNA, being translated by host ribosomes to produce essential viral proteins, including the RNA-dependent RNA polymerase (RdRp). This enzyme synthesizes a complementary −ssRNA strand, which serves as a template for generating new positive-strand RNAs that are subsequently encapsidated into new viral particles. In the case of −ssRNA viruses, the viral genome cannot be directly translated by the host machinery; thus, the resulting positive strand serves both as an mRNA for viral protein synthesis and as a template for the generation of progeny negative-strand genomes, thereby completing the viral replication cycle (Wang et al., 2020).

The high replication capacity of plant viruses renders VIGS a powerful tool for *in vivo* functional analysis of plant genes. A range of viral vectors has been successfully employed to implement VIGS in both monocot and dicot species, including Chinese Wheat Mosaic Virus (CWMV) (Yang et al., 2018; Guo et al., 2019; Kondo et al., 2022), Tobacco Rattle Virus (TRV) (Bachan and Dinesh-Kumar, 2012; Deng et al., 2021; Alter et al., 2022; Kalia et al., 2024; Liu et al., 2025), Barley Stripe Mosaic Virus (BSMV) (Bruun-Rasmussen et al., 2007; Buhrow et al., 2016; Hu et al., 2019; Harvinder and Upinder, 2022; Tayachew et al., 2024), Brome Mosaic Virus (BMV) (Ding et al., 2006, 2018; Wang et al., 2021; Singh and Mysore 2022), Cymbidium Mosaic Virus (CymMV) (Hsieh et al., 2013; Chen et al., 2019), Rice Tungro Bacilliform Virus (RTBV) (Purkayastha et al., 2010; Kant et al., 2015; Kumar *et al.,* 2022), Cucumber Mosaic Virus (CMV) (Kim et al., 2020; Liu *et al.,* 2020; Tasaki et al., 2020; He et al., 2024; Wang *et al.,* 2025), Foxtail Mosaic Virus (FoMV) (Liu *et al.,* 2016; Mei and Whitham, 2018; Tiedge et al., 2022; Beernink and Whitham, 2023), Maize Rayado Fino Virus (MRFV) (Mlotshwa et al., 2020) and Sugarcane Mosaic Virus (SCMV) (Ali et al., 2017; Mei *et al.,* 2019; Chung et al., 2022).

The delivery is achieved using methods that introduce viral vectors carrying fragments of the target gene to trigger the plant’s RNAi response (Burch-Smith et al., 2004). The most widely used approach is agroinfiltration, where leaves are infiltrated with a suspension of *Agrobacterium tumefaciens* harboring the viral vector, which can be performed manually, under vacuum, or using an airbrush to improve penetration, widely used for geminiviruses and tobamoviruses (Zhang et al., 2017; Li et al., 2024)**.** Alternatively, in mechanical infection, the plant is inoculated with purified viral particles or extracts of infected tissue. This method typically involves abrading the leaf surface to allow the virus to enter. It is most commonly used in plants with thinner leaves, such as *Nicotiana benthamiana*. The biolistic delivery (gene gun) uses DNA-coated particles shot into plant tissues, a strategy particularly useful for species that are recalcitrant to transformation (Shen et al., 2025). These methods allow efficient and transient gene silencing and have recently been expanded to enable virus-mediated genome editing approaches (Cisneros et al., 2025).

## Technical challenges and successful applications of VIGS in weeds

Virus-induced gene silencing (VIGS) presents several technical limitations intrinsic to the method itself. Some of these limitations include: i) the viral vector compatibility with the host plant species, which determines efficient infection; ii) the heterogeneous dissemination of the viral vector within plant tissues, leading to uneven silencing, and iii) the current lack of scalable and precise inoculation methods suitable for field application. Such constraints affect the reliability and reproducibility of phenotypic outcomes across plant systems. 

In weed research, however, these general limitations face several technical barriers that restrict its broader implementation: i) the high genetic variability commonly observed within weed populations complicates target gene selection and may reduce silencing stability, ii) many weed species lack well-annotated genomes, remaining recalcitrant to stable genetic transformation, limiting functional validation pipelines.; iii) the vector delivery and dissemination within the host plant are often heterogeneous, resulting in uneven silencing and reduced reliability of phenotypic outcomes; iv) from an applied perspective, the translation of VIGS to field conditions remains particularly challenging, as requires the development of scalable and precise inoculation methods, which are not yet fully established, and v) the genetic variability and innate resistance mechanisms in weeds can diminish viral infection efficiency and compromise the stability of silencing. 

Collectively, while VIGS is broadly constrained by methodological factors, its application to weed biology requires additional optimization efforts for the genetic diversity and ecological context of weed species. Continued efforts are required to improve vector design, optimize delivery systems, and integrate functional genomic data to expand its applicability as a robust tool for weed control. Although the application of VIGS in weeds is still in its early stages, some successful cases have demonstrated its feasibility and value as a functional genomics tool. Optimized VIGS in *Solanum nigrum* has been used to investigate gene function related to plant defense (Hartl et al., 2008). Using Tobacco rattle virus (TRV), it was demonstrated that leucine aminopeptidase plays a critical role in resistance against herbivores, while also highlighting limitations associated with empty vector controls in VIGS experiments.

In *Lolium temulentum*, VIGS using the BSMV vector has been successfully applied to study gene function, demonstrating that transient knockdown of endogenous genes can be achieved in this species (Martin et al., 2013). The study showed that VIGS produces visible phenotypic changes, confirming effective gene silencing, and established the feasibility of using virus-mediated approaches for functional genomics in ryegrass. For functional genomics in the parasitic plant *Striga hermonthica*, a VIGS system has been established through the TRV vector (Kirigia et al., 2014). This system enables efficient knockdown of endogenous genes, allowing the study of gene function in a species that is otherwise difficult to manipulate genetically. A Foxtail mosaic virus (FoMV)-based vector has been developed for VIGS in maize (*Zea mays*), enabling efficient knockdown of endogenous genes such as *phytoene desaturase*, *lesion mimic22*, *iojap*, and *brown midrib3* (Mei et al., 2016). The FoMV vector establishes systemic infection not only in maize inbred lines but also in sorghum (*Sorghum bicolor*) and green foxtail (*Setaria viridis*), demonstrating its broad applicability as a functional genomics tool in monocot plants. 

Virus-mediated transient expression methods, including Virus-Induced Gene Silencing (VIGS) and Virus-Mediated Overexpression (VOX), have been successfully applied to study gene function in black-grass (*Alopecurus myosuroides*) (Mellado-Sánchez et al., 2020). These approaches enable targeted gene knockdown and overexpression, allowing genotype-specific modulation of gene expression and assessment of resulting changes in herbicide resistance. Heterologous VIGS has been applied in *Ipomoea purpurea*, a species closely related to the Solanaceae, to study gene function. The approach demonstrated effective silencing of target genes, providing a valuable tool for functional genomics in non-model species (Borna, 2023). Annan et al. (2024) developed a Barley Stripe Mosaic Virus (BSMV)-based system for functional genomics in both cultivated oat (*Avena sativa*) and wild oat (*A. fatua*), including populations with multiple herbicide resistance (MHR) (Annan *et al.,* 2024). Here, it was demonstrated that BSMV can achieve high silencing efficiency (48-100%) of endogenous genes such as *phytoene desaturase* (PDS), producing the characteristic bleaching phenotype, and can also support heterologous protein expression (e.g., GFP). 

These studies have provided proof-of-concept evidence that VIGS can be used to transiently silence key genes in weed species, enabling the investigation of gene function and the validation of targets potentially associated with herbicide resistance, growth, development, and stress adaptation. Such advances not only underscore the adaptability of VIGS beyond model and crop plants but also pave the way for its consideration as an innovative component in integrated weed management strategies.

VIGS has emerged as a promising approach for the functional characterization of genes associated with weed growth, adaptation, and herbicide resistance. Current studies suggest that the efficiency of VIGS in weeds is highly species-dependent, with some taxa displaying greater resistance to gene silencing, likely due to their genetic plasticity and robust antiviral defense mechanisms (Zulfiqar et al., 2023). Notably, several weeds that are particularly problematic in agriculture due to their evolved herbicide resistance also appear to be challenging targets for VIGS. Examples include: i) *Conyza* spp. (Horseweed), characterized by multiple resistance mechanisms involving enhanced metabolism and reduced herbicide translocation; ii) *Digitaria insularis* (Sourgrass), which exhibits metabolic robustness contributing to herbicide resistance and antiviral defenses may limit viral replication and systemic spread, possibly reducing susceptibility to VIGS; iii) *Lolium* spp. (ryegrass), known for diverse and overlapping metabolic and genetic resistance mechanisms that complicate control by external agents, may also restrict viral infection and RNA accumulation, leading to reduced and variable VIGS efficiency; and (iv) *Amaranthus palmeri*, a species with widespread multiple resistance in some populations, where gene silencing strategies have been explored to reverse resistance, albeit still at an experimental stage. These species are highlighted for their economic impact and resistance patterns, making them priority candidates for research and application of VIGS as an integrated weed control tool with high ecological and economic impact, dominant presence in agricultural systems, and notable herbicide resistance.

Overall, weeds resistant to herbicides targeting key enzymes such as ALS, EPSPS, and ACCase, particularly those with multiple resistance mechanisms encompassing metabolic, morphologic, and genetic adaptations, are likely to be less responsive to VIGS-mediated silencing. Nevertheless, the ultimate effectiveness remains dependent on the efficiency of the viral vector and the specificity of the targeted gene. 

## Conclusions and final remarks

Virus-Induced Gene Silencing holds promise as a valuable and powerful reverse-genetics approach for functional studies in weedy species, enabling transient and targeted knockdown of genes without requiring stable transformation systems (Burch-Smith et al., 2004). This is particularly advantageous for species such as *Lolium spp.* and *Digitaria insularis*, which are genetically recalcitrant and display significant metabolic plasticity (Gaines et al., 2020). By silencing genes involved in herbicide detoxification, such as cytochrome P450 monooxygenases (CYP81A, CYP72A), glutathione S-transferases (GSTF1, GSTU2), and UDP-glycosyltransferases, VIGS enables functional dissection of non-target-site resistance mechanisms (Yuan et al., 2007; Délye, 2013). In addition, targeting genes in growth regulation pathways (e.g., GA20-oxidases, AUX/IAA repressors) or secondary metabolite biosynthesis (e.g., benzoxazinoids, phenylpropanoids, terpenoids) can clarify the molecular basis of competitive ability and allelopathy (Macías et al., 2007). Such insights are critical for understanding how weeds integrate metabolic adaptation, stress resilience, and phenotypic plasticity to persist under diverse agronomic practices, ultimately supporting the design of more effective and sustainable Integrated Weed Management strategies.

Although VIGS and related RNAi-based approaches have been widely promoted as promising tools for weed management, some studies present important critical perspectives regarding their practical feasibility. Much of the optimism surrounding RNAi technologies derives from promising results obtained under controlled laboratory conditions instead of consistent validation under field environments (Panozzo et al., 2025). An important concern is the occurrence of off-target effects, in which small interfering RNAs derived from the viral construct may silence unintended transcripts sharing partial sequence similarity with the target gene. To overcome this barrier, some bioinformatics support for target selection assists in selecting optimal target regions for VIGS, particularly to minimize off-target effects, avoiding conserved domains shared with crops, and improving silencing specificity (Fernandez-Pozo et al., 2015). In addition, the viral infection itself can induce physiological symptoms such as chlorosis, mosaic patterns, or growth alterations, which may overlap with or mask the phenotypes associated with gene silencing (Wu et al., 2011). Some critical limitations remain: variable silencing efficiency among diverse weed species, limited understanding of RNA uptake and systemic movement in non-model plants, significant obstacles related to delivery strategies and molecule stability, and the economic feasibility of large-scale applications (Zabala-Pardo et al., 2022).

Beyond its applications in weed biology, VIGS is a versatile tool for elucidating the genetic determinants of plant immunity and stress signaling (Senthil-Kumar and Mysore 2011). Targeting key components of immune perception, such as pattern-recognition receptors (FLS2, EFR) and nucleotide-binding leucine-rich repeat receptors (RPM1, RPS2), or downstream signaling hubs like MAPKs (MPK3/6) and WRKY transcription factors, allows detailed functional analysis of pathogen recognition and defense activation (Bigeard et al., 2015). VIGS also enables exploration of hormonal crosstalk by silencing regulators in the salicylic acid (ICS1, NPR1), jasmonic acid (COI1, MYC2), and ethylene (EIN2) pathways, revealing trade-offs between growth and immunity. The mechanistic knowledge gained from these studies will be instrumental for breeding programs aiming at durable resistance, designing integrated disease management strategies, and reducing reliance on chemical inputs while improving the ecological sustainability of crop production systems (Dangl et al., 2013).

## Data Availability

 This article is a review of published literature. No new datasets were generated or analyzed during the current study.
